# Advantages of optical coherence tomography in assessing retinal astrocytic hamartomas in children with tuberous sclerosis complex

**DOI:** 10.3389/fmed.2026.1796812

**Published:** 2026-04-07

**Authors:** Guorui Dou, Ziyi Zhou, Jiaxing Sun, Zifeng Zhang, Chunyan Wang, Dongjie Sun, Jinting Zhu, Yanni Zhu, Hao Zhang, Qihua Wang, Manhong Li, Yanchun Deng, Yusheng Wang

**Affiliations:** 1 Department of Ophthalmology, Eye Institute of Chinese PLA, Xijing Hospital, Fourth Military Medical University, Xi’an, China; 2 Department of Neurology, Xijing Hospital, Fourth Military Medical University, Xi’an, China

**Keywords:** ocular disease, ocular imaging, optical coherence tomography, retinal astrocytic hamartomas, tuberous sclerosis

## Abstract

**Purpose:**

Retinal astrocytic hamartomas (RAHs) are benign tumors that are mostly associated with a rare systemic disorder called tuberous sclerosis complex (TSC). In the clinical diagnosis of TSC, RAH has been recognized as a major feature that supports a definite diagnosis. Here, 60 patients (120 eyes) with TSC underwent a complete ophthalmologic examination and multimodality imaging using fundus photographs, spectral-domain optical coherence tomography (SD-OCT), and infrared images.

**Methods:**

Admission SD-OCT data were retrieved and utilized for analysis. Receiver operating characteristic (ROC) curve analysis was performed to calculate the cutoff value.

**Results:**

A total of 117 hamartomas were detected in 45 patients, with a mean age of 12 years. On OCT imaging, RAHs appeared as gentle or protruding lesions of varying heights (mean thickness 314 mm, ranging between 71 and 974 mm).

**Conclusion:**

For RAHs that could not be detected on fundus photographs or by ophthalmoscopy, OCT demonstrated excellent capability in revealing these lesions, particularly smaller tumors (MT < 271 mm). These results suggest that OCT can improve the detection of hamartomas and contribute to the diagnosis of TSC.

## Highlights

- What is known: Retinal astrocytic hamartomas are a common ocular manifestation of tuberous sclerosis complex and are traditionally detected using fundus photography and ophthalmoscopy.
- What is new from this research: Spectral-domain optical coherence tomography significantly improves the detection of retinal astrocytic hamartomas, especially small or subtle lesions that are not visible on fundus photographs.
- Clinical implication: A maximal tumor thickness of less than 271 μm on OCT is a useful cutoff for identifying lesions likely to be missed by conventional ophthalmoscopy, supporting the integration of OCT into routine ophthalmic screening for TSC.
- Recommendation for practice: Multimodal imaging combining SD-OCT and infrared reflectance should be considered a first-line diagnostic approach for the comprehensive evaluation and monitoring of retinal involvement in tuberous sclerosis complex.

## 1 Introduction

Tuberous sclerosis complex (TSC) is an autosomal dominantly inherited disorder characterized by hamartomas that affect multiple organ systems, including the heart, kidney, brain, skin, and eyes. According to several population-based studies, the incidence rate of TSC is approximately one in 6,000 to 13,000 live births (1, 2). Retinal astrocytic hamartoma (RAH) is the most common and characteristic ocular finding in TSC patients. The prevalence of RAH associated with TSC varies widely, ranging from 3 to 87%, but is most commonly approximately 50% (3, 4). In addition, a much higher prevalence, up to 76%, has been reported by Japanese and Chinese research groups (5, 6). The presence of RAH is regarded as one of the major criteria for the diagnosis of TSC. Retinal findings in patients with RAHs are often accompanied by subependymal giant cell astrocytoma, renal angiomyolipoma, cognitive impairment, and epilepsy, indicating systemic involvement (7). Therefore, if RAH is detected on ophthalmologic examination, a systemic workup is recommended. If a patient is diagnosed with TSC based on other systemic features, a comprehensive ophthalmologic examination is required.

Previously, based on ophthalmoscopic appearance, RAH was classified into three types: flat and translucent lesions, nodular lesions with a mulberry-like appearance and signs of calcification, and transitional lesions with mixed features of the former two (8). As a non-invasive imaging technique, the advent of optical coherence tomography (OCT) has shown its ability to reveal tumor characteristics. Recent studies have demonstrated the superiority of spectral-domain OCT (SD-OCT) in detecting RAH lesions. The emergence of OCT has demonstrated its capability to display the characteristic features of tumors in cross-section with a resolution of less than 10 microns (9). Shields et al. reported that retinal astrocytic hamartoma (RAH) on OCT often exhibits posterior optical shadowing (10). The combination of SD-OCT and infrared imaging has been shown to improve the detection of hamartomas (11, 12). Based on SD-OCT imaging, Pichi (13) studied the correlation between RAH subtypes and specific systemic manifestations of TSC. Mutolo et al. further refined Pichi’s classification. They divided Type II lesions into two groups, Type IIa and Type IIb, based on the height of the tumor and intratumoral appearance (11).

The present study examined the phenotype of RAH in a large cohort of patients with TSC at Xijing Hospital, China. For the first time, information from OCT and ophthalmoscopy was combined to construct an ROC curve, aiming to determine SD-OCT’s sensitivity in detecting small lesions and investigate its usefulness in supporting the early identification of occult RAH for proper diagnosis and management of TSC patients.

## 2 Materials and methods

### 2.1 Study design, human ethics, and consent to participate

This cross-sectional study included all patients diagnosed with tuberous sclerosis complex (TSC) from the TSC China Alliance. Data were collected from patients at the Department of Ophthalmology, Xijing Hospital, Xi’an, China. The study was conducted in accordance with the tenets of the Declaration of Helsinki and was approved by the institutional review board of Xijing Hospital. All analyses involving human specimens were conducted in full accordance with institutional guidelines and were approved by the Ethics Committee of Xijing Hospital, Fourth Military Medical University (Approval No. KY20232305-C-1). Written informed consent for data acquisition and clinical examination was obtained from the patient or his/her legal guardian.

### 2.2 Patients and materials

A total of 60 patients were examined by two different ophthalmologists experienced in retinal astrocytic hamartoma (RAH) using both direct and indirect ophthalmoscopy. The number and location of tumors were recorded in the patients’ medical reports. In cases of discrepancies between the two reports, only the report identifying the greater number of hamartomas was considered. Fundus photographs were then obtained to document these tumors based on the medical reports. According to clinical classification, these tumors were divided into three groups [3]: Type I: Semitransparent, flat, grayish lesions without signs of calcification; Type II: Multinodular, calcified lesions with a mulberry-like appearance; and Type III: Transitional lesions combining features of the previous two types. All examinations were performed on awake children with the assistance of their parents or guardians, and imaging settings were adjusted according to each child’s level of cooperation and fixation ability. For uncooperative younger children, we employed additional strategies such as repeated scanning sessions and breaks to ensure image quality.

### 2.3 OCT examination

OCT imaging of these patients was performed using a SPECTRALIS SD-OCT instrument (Heidelberg Engineering, Heidelberg, Germany) according to a previous report (11). This system allows IR scans to be obtained simultaneously with registered SD-OCT images (multimodality imaging). The imaging protocol included a 30° scan angle of the posterior pole and the four quadrants. Once the lesions were identified, details of the images were acquired using line scans and the detail preset pattern (15° × 15°volume, density 30 μm). The standard for images was the high-resolution mode; however, settings for resolution, the number of frames per B-scan, and the number of B-scans per volume scan were adjusted according to the patient’s level of cooperation and fixation ability. According to previous studies, the distance from the highest peak of the anterior surface of the RAH to the pigment epithelium was defined as maximal thickness (MT, μm), and base diameter (BD, μm) was measured as the base area of the tumor from the normal retina. The location of the tumor epicenter was divided into five areas: Supratemporal, inferotemporal, superonasal, inferonasal, and macular regions. All hamartomas were measured and morphologically characterized in terms of reflective heterogeneity, OECs, tumor margins and transition (gradual or abrupt), and vitreous involvement (traction or seeding). An OCT classification for RAH, originally proposed by Pichi et al. (13) in 2016 and later modified by Mutolo et al. (11) in 2017, was adopted as follows: Type I: SD-OCT shows a flat lesion, mainly involving the retinal fiber layer, with no retinal retraction; Type IIa: SD-OCT shows an elevated, dome-shaped tumor with homogeneous internal reflectivity; Type IIb: SD-OCT shows a highly protruded lesion with heterogeneous internal reflectivity, small empty spaces or cysts, and segmented vascular calcification within the tumor; Type III: SD-OCT shows an elevated retinal mass (height>500 μm) with inner retinal calcification and clinical evidence of mulberry-like calcification; and Type IV: SD-OCT shows an elevated retinal mass (height>500 μm) with a large optically empty cavity and clinical evidence of a smooth, non-calcified inner retinal mass.

### 2.4 Data analysis

All data were analyzed using statistical software (SPSS, ver. 25; Statistical Package for the Social Sciences, IBM, Armonk, NY, USA). Continuous variables are presented as the mean ± standard deviation. Statistical evaluation was performed to compare the demographic characteristics of patients with and without RAHs using the chi-squared test or one-way ANOVA, as appropriate. An independent samples *t*-test was used to compare differences between the tumor groups. For OCT parameters, a receiver operating characteristic (ROC) analysis was performed and the area under the curve (AUC) was estimated. The Youden’s index (YI) and diagnostic odds ratio were used as measures of diagnostic accuracy to determine cutoff values. A *p*-value of <0.05 was considered statistically significant.

## 3 Results

### 3.1 Patient demographic characteristics

A total of 120 eyes from 60 patients with TSC were analyzed using fundoscopy, SD-OCT, and infrared imaging. However, seven eyes failed to complete the ophthalmic examinations due to poor coordination and fixation. RAHs were found in 45 patients (64%) and were bilateral in 51.1% (23/45) of these patients. According to historical chart records, only eight of these 45 patients had been advised or scheduled for a funduscopic examination at the time of their initial diagnosis of TSC, and only five were diagnosed with RAH. The age of the patients ranged from 2 to 21 years, with a mean age of 12 years among the 45 patients with RAH. Of these patients, 21 were female and 24 were male. Statistical analysis revealed no significant difference in age and sex between TSC patients with and without RAH. Patient demographic data are summarized in Table 1. Overall, among the 45 patients, 64 lesions were detected using fundoscopy, while 117 RAH lesions were detected using SD-OCT combined with IR.

**Table 1:** Demographic characteristics.

Characteristics	N (%)		*p-*value	OR (95% CI)
	Without RAH	With RAH		
Sex				
Male	9 (60%)	21 (46.7%)	0.371^*^	0.58 (0.18-1.9)
Female	6 (40%)	24 (53.3%)		
Age at ocular examination				
Mean	11.5	12	0.157^†^	
Median, range	12 (2–21)	10 (2–37)		
RAH laterality, no. (%)				
Unilateral, eye only		22 (48.9%)		
Bilateral		23 (51.1%)		

^*^Chi-squared analysis.

^†^One-way ANOVA.

### 3.2 Retinal findings in fundus photography

Among the 45 patients (85 eyes, with five eyes unable to complete the ophthalmic examinations), 64 RAHs were observed using fundoscopy. The presence of the retinal achromic patch (AP) was noted in only 2.3% (2/85) of the eyes. Most tumors (59; 92%) were extramacular, while only five (7.8%) were macular. Based on fundus appearance, the lesions were classified as follows: 49 were Type I (semitransparent, flat, grayish lesions confined to the retinal NFL, without signs of calcification), three were Type II (multinodular, calcified lesions with a mulberry-like appearance), and 12 were Type III (transitional lesions) (See Table 2).

**Table 2:** Characteristics in fundus images.

Characteristic	Number	Percentage (%)^*^
Type		
Type I	49	76.6
Type II	3	4.69
Type III	12	18.8
Quadrant		
Superior temporal quadrant	32	50
Inferior temporal quadrant	15	23.4
Superior nasal quadrant	5	7.8
Inferior nasal quadrant	7	10.9
Macular area	5	7.8
Location		
Macular area	5	7.8
Macular to the equator	54	84.4
Equator to the ora serrata	5	7.8

^*^Calculated based on 64 funduscopically visible lesions.

The most common morphological type of hamartoma in our series was Type I, which was observed in 49 of the 64 tumors visible by fundoscopy (76.6%). Their sizes ranged from 0.25 to 2 disc diameters, and they were usually located in the posterior pole and frequently appeared to be superficial to the retinal vessels. Type II lesions were observed in only three tumors (4.69%). They were all located in the posterior pole, adjacent to the optic disc, and measured approximately 2 disc diameters. Type III lesions were observed in 12 tumors (18.8%). In all cases, these were flat hamartomas with a central area of nodular calcification.

### 3.3 OCT features of lesions

Optical coherence tomography (OCT) was performed for all patients (*n* = 60). In total, 117 RAHs were detected in 45 patients (85 eyes) using SD-OCT in combination with IR. A comparison between OCT and fundoscopy revealed OCT features that were not visible on fundus photographs. A total of 53 of these hamartomas were not identified during previous ophthalmoscopic examinations. Through OCT imaging, it was possible to identify multiple lesions with characteristic optical reflectivity qualities. All tumors appeared to be confined to the retinal layers. Typically, OCT imaging of most tumors showed thickening of the retinal nerve fiber layer, with varying degrees of compression or disruption of the normal retinal layers. Partial or complete shadowing could be viewed in the subjacent structure of the hyperreflective lesions. A total of 83 (71.8%) lesions showed homogeneous reflectivity, while 29 tumors (24.8%) demonstrated varying intrinsic cavitary spaces, a feature previously described by Shields as OECs. In three lesions, hyperreflective spots, presumably representing intratumoral calcification, were noted. Vitreous traction was noted in 28 lesions; vitreous seeding by retinal astrocytic hamartoma was observed in 15 eyes. We did not observe intraretinal fluid or retinal edema adjacent to the tumor in our case series. Among the 117 tumors measured by OCT, MT ranged from 71 to 974 mm (mean 314 mm), and BD ranged from 270 to 5,615 mm (mean 2,244 mm).

**Figure 1:**
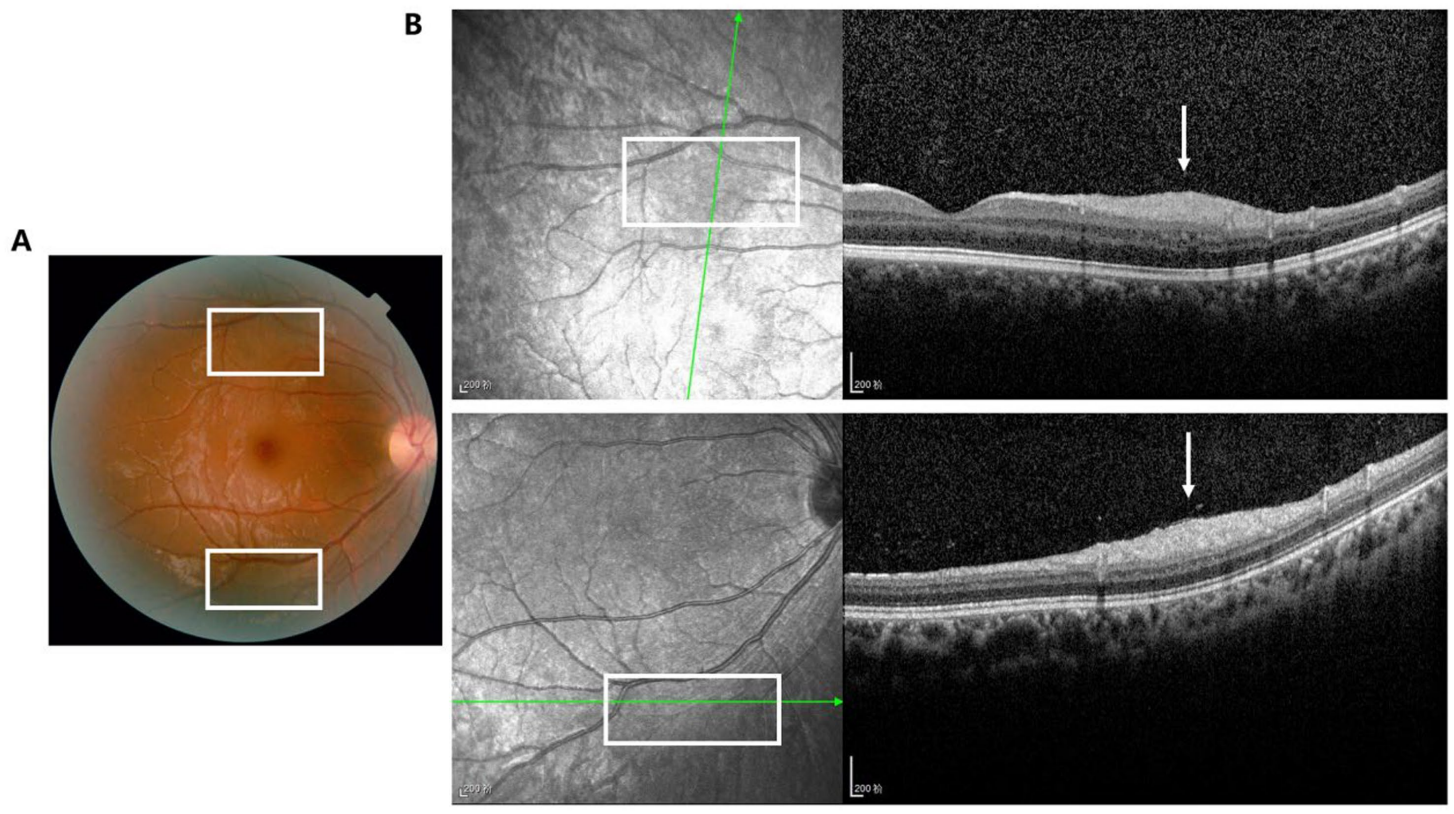
Type I RAH (white box) is characterized by a relatively flat mass with homogenous reflectivity (white arrow) in both the fundus photograph **(A)** and OCT image **(B)**. Scale bar, 200 μm.

**Figure 2:**
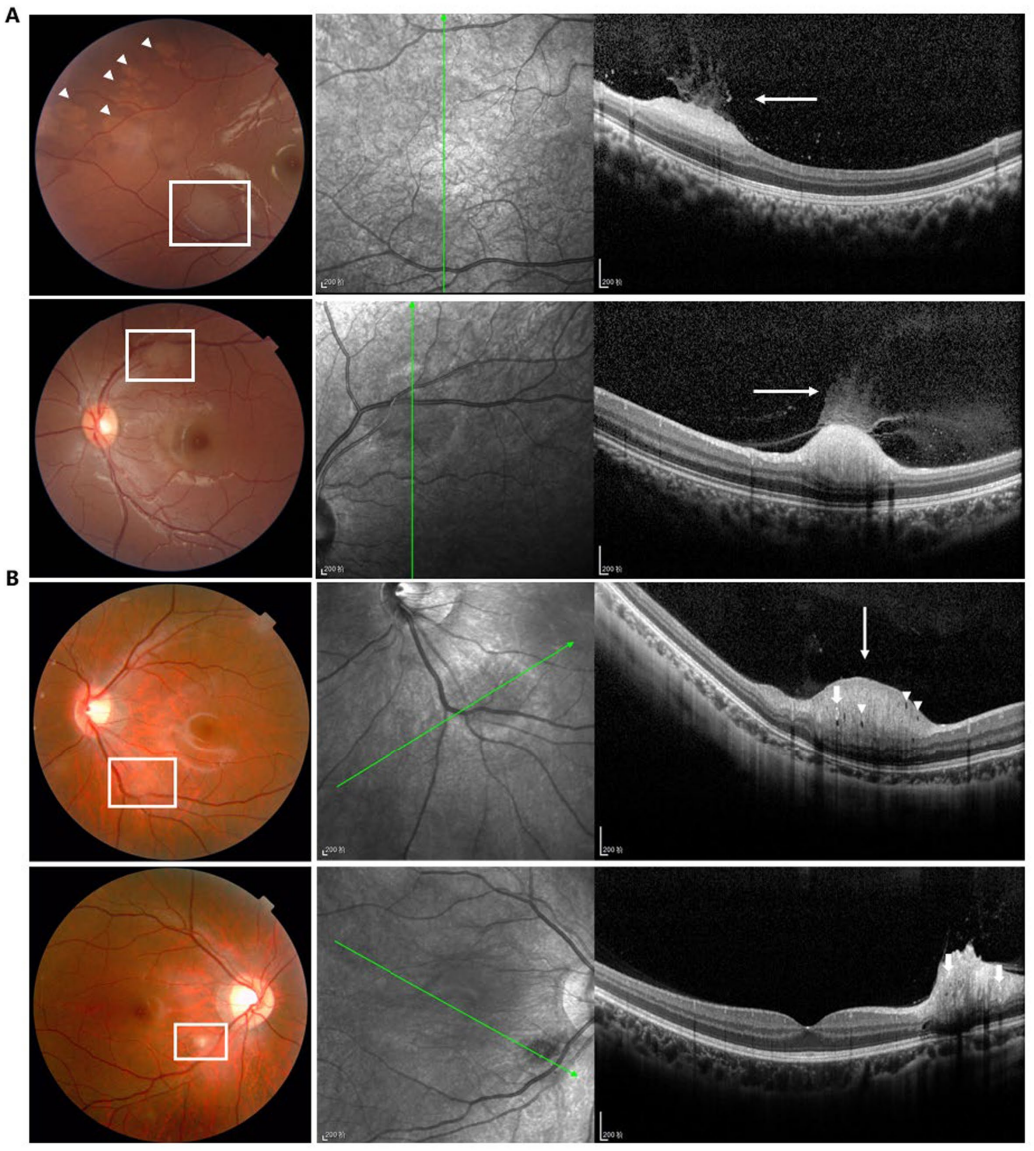
Type II RAH is characterized by an elevated, dome-shaped tumor with homogenous **(A)** or heterogeneous **(B)** reflectivity. Vitreoretinal attachment (white arrows), hyperreflective foci (bold white arrow), and moth-eaten cavities (white arrow heads) can be observed in the lesions. Scale bar, 200 μm.

**Figure 3:**
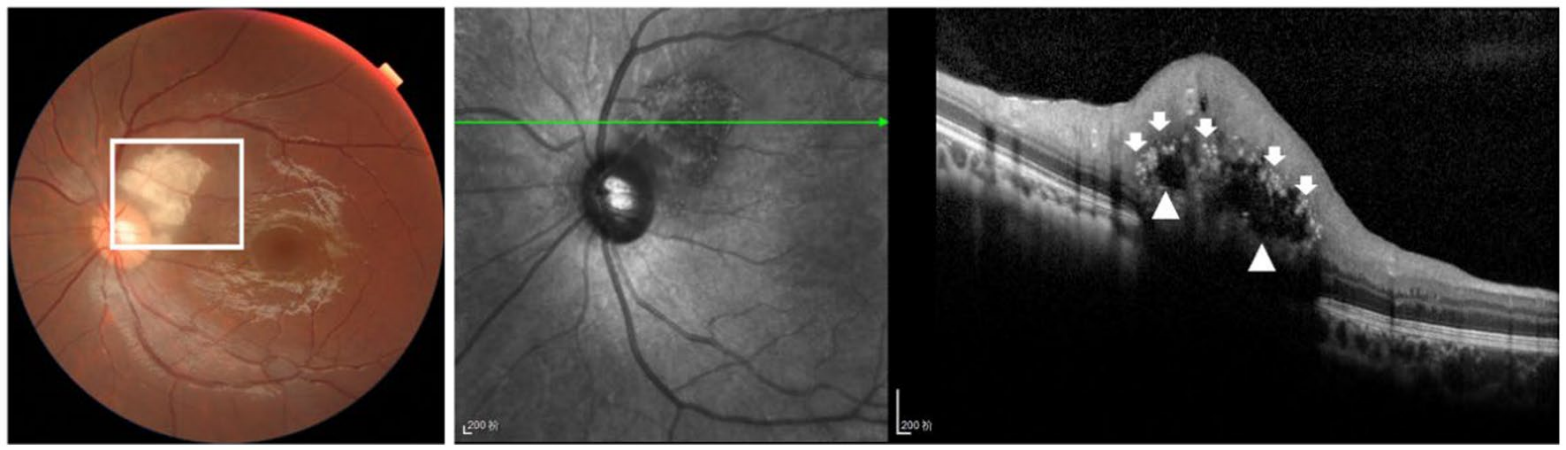
OCT image of type III RAH showing a calcified lesion with internal “moth-eaten” optical empty spaces (white arrowheads and white arrows), although calcification was clinically visible in only two lesions (white box). Scale bar, 200 μm.

**Figure 4:**
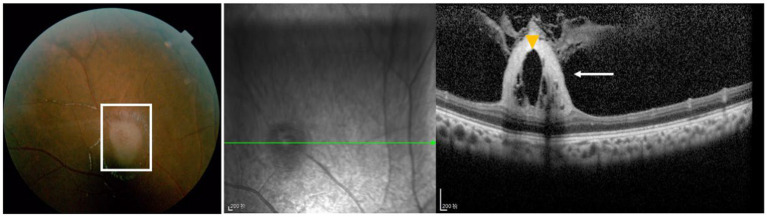
Type IV RAH is characterized by a dome-shaped tumor with a large, single cavity (yellow arrowhead) within the mass (white box in the left panel and white arrow in the right panel) in both the fundus photograph and OCT image. Scale bar, 200 μm.

**Figure 5:**
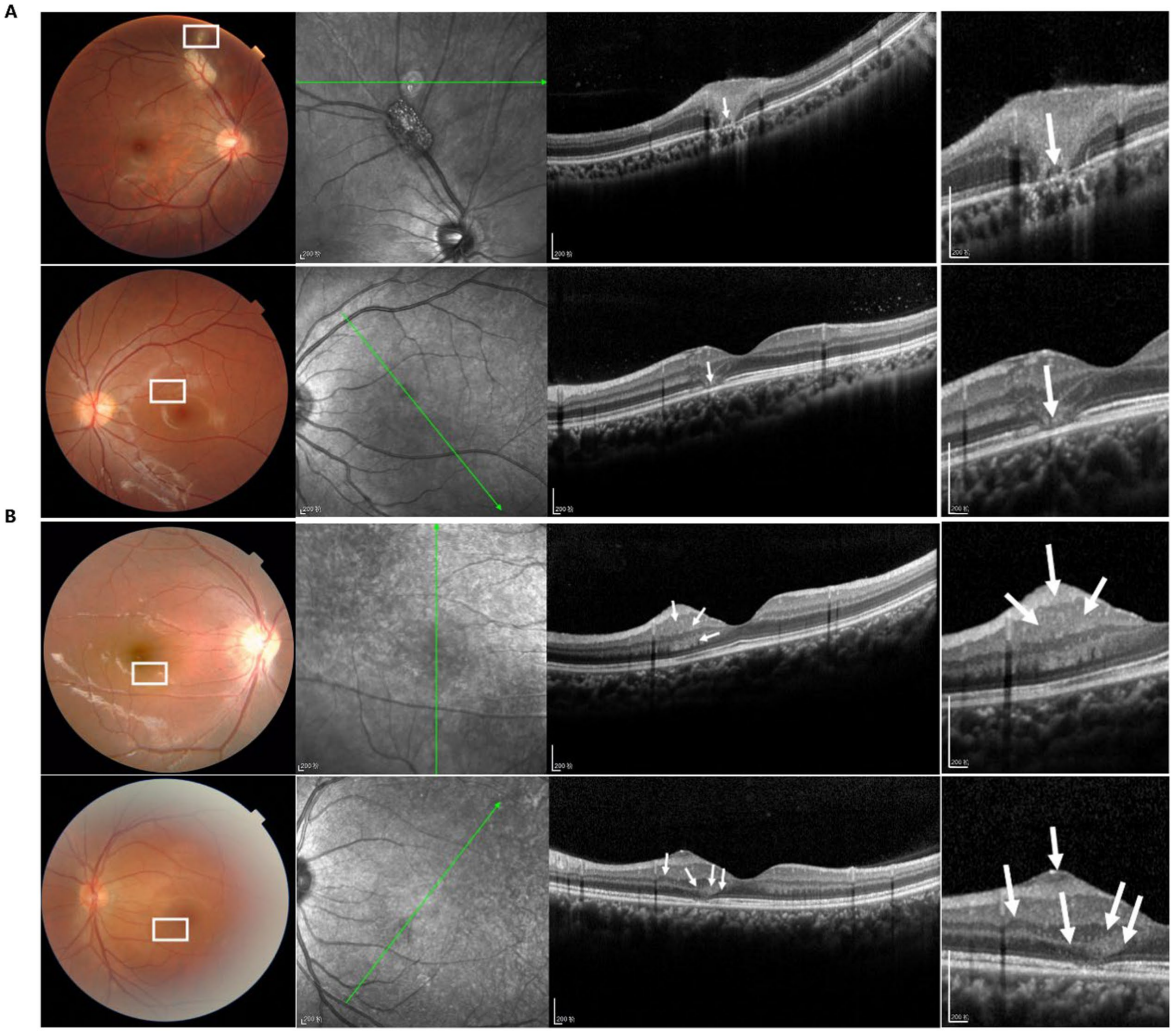
It should be noted that four lesions classified as type II did not exhibit the typical OCT features. In two lesions, the surface was mildly protruded inward toward the outer retinal layer, and the ellipsoid zone was fairly disturbed (**A**, white arrow). In the other two lesions, all retinal layers were thickened and partially disrupted (**B**, white arrow). Scale bar, 200 μm.

We also classified the SD-OCT findings of RAHs according to the classification proposed by Pichi et al. (13). A total of 40 lesions were Type I (Figures 1A,B), all characterized by a relatively flat mass with homogenous reflectivity. In total, 72 lesions were Type II, featuring an elevated, dome-shaped tumor. In these lesions, vitreoretinal attachment with homogenous reflectivity (Figure 2A) and hyperreflective foci around typical ‘moth-eaten’ cavities with heterogeneous reflectivity (Figure 2B) could also be observed. A total of three lesions were Type III (Figure 3). These lesions showed calcified features with internal “moth-eaten” optical empty spaces on OCT imaging, although calcification was clinically visible in only two lesions. In total, two lesions were classified as Type IV, characterized by a dome-shaped tumor with a large, single cavity within the mass (Figure 4A). We also subclassified the Type II lesions according to the modified classification by Mutolo et al. into Type IIa and Type IIb (Figure 5A). It should be noted that four lesions classified as Type II did not exhibit the typical OCT features. In two of these lesions, the surface was mildly protruded inward toward the outer retinal layer, and the ellipsoid zone was fairly disturbed. In the other two lesions, all the retinal layers were thickened and partially disrupted (Figure 5B). Unlike the conditions reported by Mutolo et al. (11) in our cohort, IR imaging was able to reveal the most subtle tumors. The characteristic hyporeflective appearance of RAH on IR imaging was not clearly detectable in 13 tumors (11%). The mean BD of these tumors was 2,450 ± 110 mm, and the maximal thickness was 150 ± 60 mm, presenting as gentle slopes.

**Figure 6:**
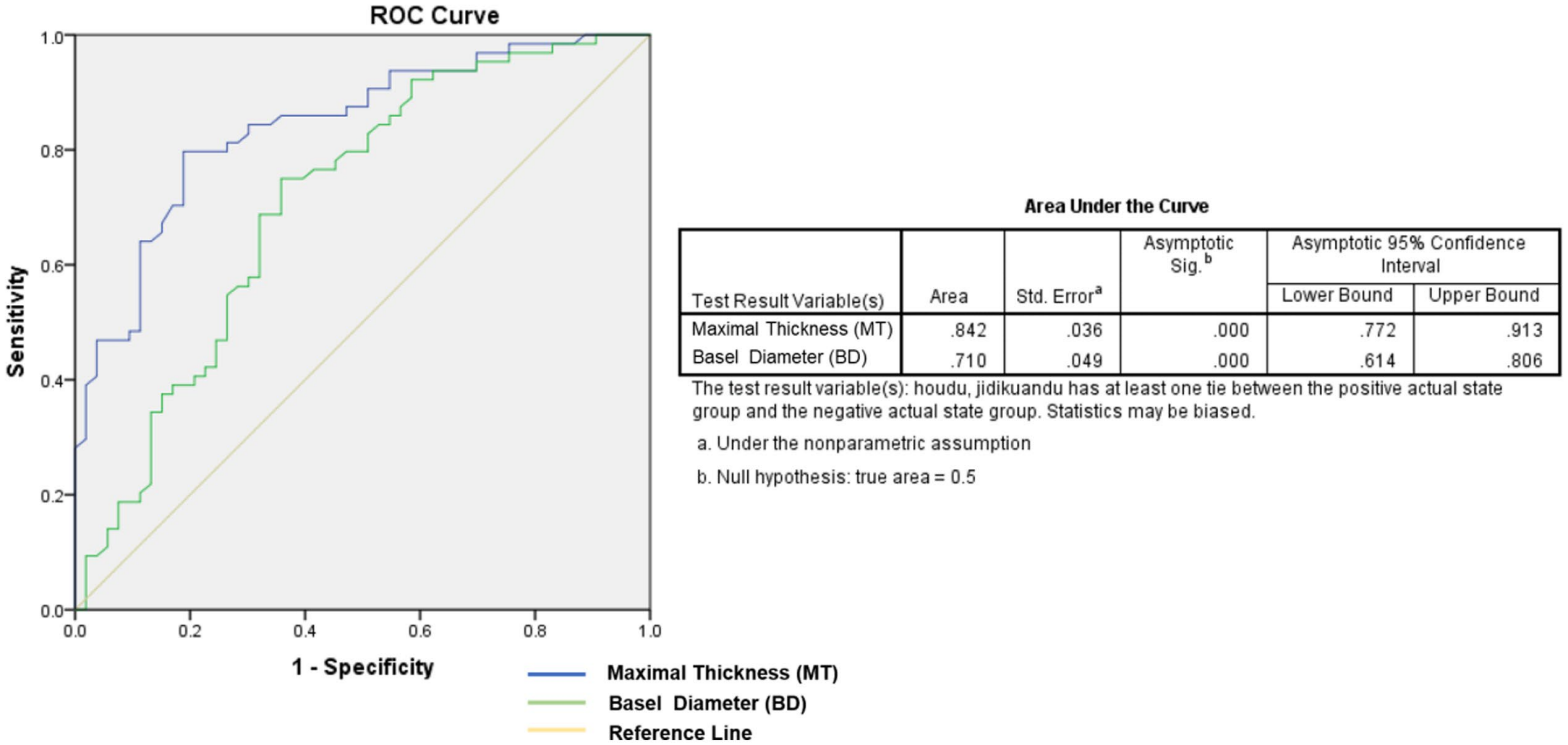
In ROC analysis evaluating the diagnostic power to discriminate tumors, MT demonstrated a higher area under the curve of 0.84 compared to 0.71 for BD.

We further divided the 117 tumors into two groups: (1) tumors visible by ophthalmoscopy, and (2) tumors detectable by OCT but not by ophthalmoscopy. The mean size of tumors visible by ophthalmoscopy was as follows: MT: 423 ± 26 mm (range, 108–974 mm) and BD: 2475 ± 134 mm (range, 560–5,430 mm); while the mean size of tumors detectable by OCT but not by ophthalmoscopy was as follows: MT: 205 ± 133 mm (range, 71–485 mm) and BD: 1741 ± 152 mm (range, 270–5,615 mm). In tumors detectable by OCT but not by ophthalmoscopy, both MT and BD were significantly smaller than those detectable by ophthalmoscopy (*p* < 0.05). In ROC analysis assessing the diagnostic power to discriminate tumors that could not be detected by ophthalmoscopy, MT demonstrated a higher area under the curve (AUC = 0.84) compared to BD (AUC = 0.71) (Figure 6). The cutoff value at 81.1% specificity was 271 mm for MT.

## 4 Discussion

Previous studies have reported that astrocytic hamartomas might occur in approximately 50% of individuals with TSC (14), with this prevalence typically based on hamartomas detected by direct or indirect ophthalmoscopy. In contrast to isolated RAHs, TSC-associated RAHs are more likely to be multifocal and involve the superior quadrant. Notably, they exhibit a markedly higher rate of complete retinal disorganization and are uniquely associated with SRF (15). In this study, we employed multimodality imaging, specifically SD-OCT, to describe the qualitative and quantitative features of 117 RAHs in patients with TSC. A total of 53 of these tumors had not been previously detected by ophthalmoscopy performed by two ophthalmologists. The higher prevalence (75%, 45/60 TSC patients) observed with SD-OCT compared to 55% detected by ophthalmoscopy (33/60 patients) further supports previous findings (11, 12), indicating that the true prevalence of RAH in TSC may be significantly higher than 50% when assessed using SD-OCT.

SD-OCT is more likely to detect or confirm semitransparent RAH lesions that may be overlooked by ophthalmoscopy alone. We divided the 117 tumors into two groups: Those visible by ophthalmoscopy and those not detectable by ophthalmoscopy. Comparison and analysis of the maximal thickness (MT) and basal diameter (BD) of the tumors between the two groups revealed that, in addition to obvious anatomical changes, tumor maximal thickness is a critical factor for determining if a lesion can be detected by ophthalmoscopy. Although tumor BD also showed a significant difference between the groups, MT showed a larger area under the receiver operating characteristic curve (AUROC = 0.842) compared to BD (0.710), suggesting that MT is a more appropriate reference parameter. A cutoff value of <271 μm was effective in distinguishing tumors not visible by ophthalmoscopy. Given that ophthalmoscopy is commonly used as a first-line examination, we recommend incorporating OCT for enhanced clinical relevance in the diagnosis and characterization of TSC patients.

Studies have demonstrated that ultra-widefield scanning laser ophthalmoscopy (UWF-SLO) achieves a higher detection rate for tuberous sclerosis complex (TSC)-associated retinal astrocytic hamartomas (RAHs) compared to conventional color fundus photography (CFP). Given the superior penetrative capability of laser-based imaging, it is recommended to combine UWF-SLO with conventional CFP and optical coherence tomography (OCT) for a comprehensive evaluation of posterior pole RAHs, thereby enhancing lesion identification and facilitating long-term disease monitoring (16). In addition, emerging evidence suggests that multimodal imaging significantly improves diagnostic accuracy for early-stage, atypical retinal astrocytic hamartomas. Notably, these lesions typically require no specific intervention, as spontaneous tumor regression, along with the resolution of associated edema and exudates, has been well documented. However, close surveillance through serial fundoscopic examinations, OCT, and OCT angiography (OCTA) is strongly recommended prior to complete calcification to monitor disease progression (17).

High-resolution SD-OCT scans can reveal the most accurate tumor structures and unique characteristics. On OCT imaging, RAHs appear as hyperreflective tumors with a gradual or abrupt transition from the normal retina and are likely to originate from the nerve fiber layer. Partial or complete posterior shadowing might be present, but not in all cases. A total of 15 eyes showed vitreous seeding from the tumor surface, a phenomenon previously described and presumed to represent tumor cells rather than inflammatory cells (18, 19). Based on SD-OCT, OESs, first recognized by TD-OCT (9), represent calcifications or cavities within RAHs. In accordance with findings from Sheilds (10), we also witnessed a relationship between the number of OESs and their increasing size in our case series. OESs are a critical feature in OCT classification. For instance, OESs associated with calcified lesions classified as Type III have been linked to subependymal astrocytoma, while a large single cavity, corresponding to Type IV, may be indicative of pulmonary lymphangiomyomatosis. However, a Japanese study did not observe this correlation, which may be due to differences in the follow-up period, patient age, or ethnicity. Meanwhile, OCT is particularly valuable in differentiating RAH from retinoblastoma (RB), as RAH typically shows characteristic features, such as a gradual transition from the normal retina, hyperreflectivity, and empty cavities, that are distinct from the typical findings in RB.

The study also has some limitations. In our case series, using the OCT classification proposed by Pichi and Mutolo (11, 13), we subclassified tumors into Type I, Type IIa, Type IIb, Type III, and Type IV. However, we noted that intratumoral characteristics were not relevant to tumor thickness. Therefore, OCT-based RAH classification requires further refinement through additional studies. In addition, we did not verify this predictive capacity of the OCT classification, as 50% of our patients were under 10 years old and 80% of our patients were under 18 years old. This study used a cross-sectional design, with ocular examinations performed at a single time point for each individual. As many systemic manifestations of TSC are age-dependent, pulmonary involvement (LAM) occurs in up to 35–80% of women with TSC by age 40, while structural brain abnormalities (subependymal giant cell tumors, SGCT) require ongoing surveillance with MRI until approximately 25 years of age. Given the dynamic progression of systemic manifestations in TSC, a longitudinal evaluation would have been ideal to determine whether small, non-visually detectable hamartomas increase in size over time and become detectable by ophthalmoscopy or whether some lesions remain small and otherwise go undetected without OCT. Under these circumstances, we plan to analyze these correlations in the future as additional follow-up data become available for these growing patients. Genetic testing is essential for the diagnosis of TSC-associated RAHs. In the present study, not all patients underwent genetic testing, which represents a limitation that needs to be addressed in future research. Moreover, the absence of a control group with non-TSC retinal lesions limits our ability to assess the specificity of the OCT features described above.

Multimodality imaging using SD-OCT combined with IR images has been proposed in previous studies (11, 12). Changes in the background reflectivity of IR images are related to the presence of RAHs on SD-OCT scans. In our cases, 13 tumors (11%) were not clearly detectable by IR imaging. Interestingly, unlike the small, limited-dimension tumors (mean MT: 268 mm, mean BD: 0.4 mm) reported by Mutolo, these lesions exhibited a wide base with low elevation (MT: 150 ± 60 mm, BD: 2450 ± 110 mm). The lesions of this shape have a density similar to that of normal tissue, thus they do not show any particular features in infrared imaging. This hypothesis, however, should be investigated and confirmed in future studies. There is no doubt that SD-OCT combined with IR imaging could facilitate regular, detailed follow-up, enabling assessment of the development of retinal hamartomas over time. Based on our study, we strongly advocate the use of SD-OCT combined with IR imaging as the preferred modality for the diagnosis and surveillance of RAHs in patients with TSC.

Previous studies have examined the prevalence of ophthalmic manifestations of TSC in developed countries, including the USA, UK, and Japan (5, 7, 20). A study from Sweden reported that ophthalmological records were available for 50 of the 52 children diagnosed with TSC in the region. The mean age at the last visit was 12.4 years (21). In addition, RAH may also occur in the context of neurofibromatosis (NF). Further studies are needed to determine whether the features of NF-associated RAH are distinct from those associated with TSC or with isolated RAH. A recently published study reported the clinical phenotype of RAH in Chinese TSC patients (6). In that study, all TSC patients were referred to ophthalmologists for the evaluation of RAH. However, not all TSC patients, at least in China, are aware of ocular involvement in this multisystem disorder. In our study, only a few patients had been advised to undergo a funduscopic examination at the time of their initial TSC diagnosis, and none had received annual ophthalmologic follow-up. It should be noted that, according to their historical chart records, only eight of these 60 patients had been advised or scheduled for a funduscopic examination at the time of their initial TSC diagnosis. Although it is already widely accepted that a complete check-up and continued surveillance of TSC patients are needed to assess organ dysfunction, new lesions, or the growth of previously identified lesions (22), none of the patients in this study had undergone annual ophthalmologic examinations during their prior routine follow-up. Compared to the brain and kidneys, the eyes, skin, and teeth appear to be the most frequently overlooked. If a patient is diagnosed with TSC based on other systemic features, a comprehensive ophthalmologic examination is necessary. Although most retinal hamartomas are non-progressive and rarely affect vision (1), some cases have reported progression, including subretinal fluid, neovascularization, and exudative retinal detachment. Treatment options, such as intravitreal anti-vascular endothelial growth factor therapy, triamcinolone acetonide, laser photocoagulation, or photodynamic therapy, may have limited effectiveness (23–27). For patients with TSC-associated RAHs complicated by vitreous hemorrhage, studies have suggested that vitrectomy combined with sirolimus may be the better choice, with intravitreal anti-VEGF injection serving as an adjunctive therapy (28).

In summary, based on our observations, multimodal imaging—especially SD-OCT—plays a significant role in the diagnosis of RAH, which can contribute to the confirmation of TSC. In such cases, medical staff should keep in mind that it is their responsibility to adequately inform newly diagnosed patients and their parents about the multisystem nature of TSC, as well as the importance of comprehensive ophthalmic evaluation.

## Data Availability

The original contributions presented in the study are included in the article/supplementary material, further inquiries can be directed to the corresponding authors.
